# Efficacy and adverse reactions of perampanel in the treatment of epilepsy in children

**DOI:** 10.3389/fneur.2022.924057

**Published:** 2022-07-27

**Authors:** Dan Li, Shaoping Huang, Xueying Wang, Lin Yang, Tingting Song

**Affiliations:** ^1^Department of Pediatrics, The Second Affiliated Hospital of Xi'an Jiaotong University, Xi'an, China; ^2^Fifth Department of Pediatrics, Northwest Women's and Children's Hospital, Xi'an, China

**Keywords:** perampanel, epilepsy, children, efficacy, adverse reactions

## Abstract

**Objective:**

To observe the clinical effect and adverse reactions of perampanel in the treatment of epilepsy in children.

**Methods:**

A retrospective analysis was performed on 83 children with epilepsy who were treated with perampanel in the Department of Pediatric Neurology, Second Affiliated Hospital of Xi'an Jiaotong University from April to August 2021. The treatment status, prognosis and adverse reactions were followed up. The effective rates of different age groups, different seizure types and epilepsy syndromes, and different treatment methods were statistically analyzed. The effective rate and adverse reactions of all patients were statistically analyzed.

**Results:**

The overall effective rate of perampanel in the treatment of epilepsy was 62.03%, and there was no significant difference in the effective rate of perampanel in the treatment of epilepsy in patients of different ages (*P* > 0.05). The effective rates of perampanel in the treatment of focal seizures and generalized seizures were 60.38% and 65.38%, and the effective rates of benign childhood epilepsy with centrotemporal spikes (BECT), BECT combined with electrical status epilepticus during sleep (ESES) and frontal lobe epilepsy (FLE) were 88.89, 72.73, and 66.67%. The effective rates of monotherapy and combination therapy were 88.88 and 58.57%, respectively. The above statistical differences were not statistically significant (*P* > 0.05). In addition, the adverse reaction rate of perampanel treatment was 16.45%, including irritability, drowsiness, dizziness, nausea, vomiting and abnormal liver function.

**Conclusion:**

Perampanel has a high efficiency and controllable adverse reactions in the treatment of childhood epilepsy. This drug can be used as a reliable choice for long-term use in the treatment of epilepsy in children.

## Introduction

Epilepsy is a neurological disorder characterized by highly synchronized abnormal firing of neurons, with a global incidence of ~61.4/100,000 person-years (1, 2). Recurrent epileptic seizures can seriously affect the neurobiological, cognitive, psychological, and social functioning of patients (1, 2). There are many ways to treat epilepsy in clinical practice, and drugs are still the first choice for antiepileptic treatment. Studies have shown that 55%−68% of patients can achieve long-term remission of epilepsy with reasonable treatment, but about one-third of patients with epilepsy still have uncontrolled seizures and develop refractory epilepsy (3, 4).

The advent of new antiepileptic drugs in recent years has provided favorable conditions for the effective treatment of epilepsy. In 2012, the third-generation new anti-epileptic drug perampanel was approved in Europe and the United States as partial-onset epilepsy (with or without secondary generalized seizures) and primary generalized tonic in patients with epilepsy aged 12 years and older Indications for the add-on treatment of convulsive seizures epilepsy with an increasing age range (5). In 2018, the Food and Drug Administration (FDA) approved perampanel as a monotherapy and adjuvant therapy for focal epilepsy (with or without secondary generalized seizures) in children 4 years of age and older (6). However, the current domestic and foreign data on the clinical efficacy and adverse reactions of perampanel in the treatment of childhood epilepsy are still limited. This article aims to observe and analyze the efficacy and adverse reactions of perampanel as a single drug or add-on therapy in the treatment of epilepsy in children, in order to provide more data support for the application of perampanel in children with epilepsy.

## Methods

### Patients

A retrospective analysis was performed on 83 children with epilepsy who were treated with perampanel in the Department of Pediatric Neurology, Second Affiliated Hospital of Xi'an Jiaotong University from April to August 2021. The enrolled patients were followed up by telephone, outpatient or inpatient, and their treatment status was recorded. Among the 83 follow-up patients, four were lost to follow-up, and 79 patients completed the follow-up. All the patients who completed the follow-up were followed up for more than 6 months, of which eight were followed up for more than 12 months, and 71 were followed up for more than 6 months. This research protocol complies with the relevant requirements of the World Medical Association Declaration of Helsinki, and all patients have signed the informed consent form. This study was approved by the Ethics Committee of the Second Affiliated Hospital of Xi'an Jiaotong University (approval number: 2022027).

### Inclusion and exclusion criteria

#### Inclusion criteria

➀ Children under the age of 14; ➁ patients diagnosed with childhood epilepsy; ➂ patients treated with perampanel; ➃ patients who agreed and signed the emotional consent form.

#### Exclusion criteria

➀ Patients over 14 years of age and older; ➁ pilepsy patients not treated with perampanel; ➂ patients with severe other diseases affecting follow-up; ➃ patients who did not agree to be included in this study.

### Treatment methods and observation indicators

All patients were diagnosed with epilepsy after admission. According to the different methods of medication in their treatment, they were divided into two groups: monotherapy (nine patients) and combination therapy (70 patients). Monotherapy: the initial dose of perampanel was (0.5–2.0) mg/day, and the maintenance dose was (1.5–12.0) mg/day, with an average of 4.15 mg/day. The treatment time is more than 6 months. The combined treatment plan: adding perampanel to the anti-Seizure Medication drugs (ASMs) treatment. The initial dose of perampanel was (0.5–2.0) mg/day, and the maintenance dose was (1.5–12.0) mg/day, with an average of 4.15 mg/day. The treatment time is more than 6 months. ASMs drugs in this study included valproic acid (VPA), levoacetacetam (LEV), topiramate (TPM), ocazepine (OXC), clonazepam (CZP), nitrazepam (NZP), carbamazepine (CBZ), Lacosamine (LCM) and lamotriazine (LTG). In the combined treatment group, five patients had one kind of ASMs, 10 patients had two kinds of ASMs, 35 patients had three kinds of ASMs, 17 patients had four kinds of ASMs, five patients had five kinds of ASMs.

All patients were followed up and the treatment status, prognosis and adverse reactions were recorded. Drug-related adverse reactions were collected during administration and follow-up. The treatment effective response rate of epilepsy was evaluated as a reduction of ≥50% than baseline (monthly evaluated) or seizure freedom. The treatment response rates of different seizure types and epilepsy syndromes were statistically analyzed. The response rates of monotherapy and combination therapy were compared and statistically analyzed.

### Statistical methods

SPSS 25.0 software was used for data analysis. Normally distributed measurement data were expressed as mean ± standard deviation (SD), while non-normally distributed measurement data were expressed as median (interquartile range), and the comparisons were examined by Student-*t*-test and Mann–Whitney test (non-parametric distribution). The categorical data were expressed as n (%), and the differences between the two groups were examined by chi-square analysis or Fisher's Exact Test. *P* < 0.05 means the difference is statistically significant.

## Results

### General information of patients

A total of 79 patients were collected, including 49 males and 30 females, with a male-to-female ratio of 1.63:1. The average age was (10.61 ± 4.38) years, and the weight was (33.08 ± 16.14) kg. Age of onset: the youngest was 2 days after birth, the oldest was 13 years old, the interquartile range of onset age was (0.5–5.5) years old, and the median onset age was 2.0 years old. The age of starting perampanel treatment: <4 years in six cases, 4–12 years in 49 cases, >12 years in 24 cases, with an average age of (10.23 ± 4.41) years. The initial dose of perampanel was (0.5–2.0) mg/day, and the maintenance dose was (1.5–12.0) mg/day, with an average of 4.15 mg/day (Table 1).

**Table 1:** General information of patients.

**Index**	**Value**	**Monotherapy**	**Combination therapy**	χ^2^**/*****t***	***P*** **-value**
Male	49 (62.03%)	4	45	1.333	0.248
Female	30 (37.97%)	5	25		
**Age (year)**					
<4	6 (7.59%)	2	4	4.138	0.126
4 12	49 (62.03%)	6	43		
>12	24 (30.38%)	1	23		
Weight (kg)	33.08 ± 16.14	27.44 ± 11.59	33.81 ± 16.56	−1.116	0.268
Average age (year)	10.61 ± 4.38	7.94 ± 3.48	10.41 ± 4.52	−1.580	0.118
Onset age (year)	3.32 ± 3.29	5.54 ± 4.49	3.06 ± 3.00	2.211	0.030
Initial dose of perampanel (mg/day)	0.5 2.0	0.5 2.0	0.5 2.0	0.000	>0.999
Maintenance dose of perampanel (mg/day)	1.5 12.0	1.5 12.0	1.5 12.0	0.000	>0.999

### Comparison of therapeutic effects of perampanel in different age groups

A total of 49 (62.03%) patients achieved effective response after treatment. In group under the age of 4 years old, four (66.67%) person achieved effective response. In group between 4–12 years old, 29 (59.18%) achieved effective response. In group over 12 years old, 16 (66.67%) achieved effective response. There was no significant difference in effective rate among different age groups (*P* > 0.05; Table 2)

**Table 2:** Comparison of effective rates of different types of perampanel.

**Index**	**Total number**	**Effective number of cases**	**Effective rate**	**χ^2^**	***P*** **-value**
**Age (year)**					
<4	6	4	66.67%	0.442	0.802
4 12	49	29	59.18%		
>12	24	16	66.67%		
**Epileptic seizure type**					
Focal seizure	53	32	60.38%	0.186	0.667
Generalized seizure	26	17	65.38%		
**Epileptic syndrome**					
BECT	9	8	88.89%	5.483	0.019
BECT combine ESES	11	8	72.73%		
FLE	3	2	66.67%		
CSWS	2	1	50.00%		
TLE	4	2	50.00%		
Dravet	9	4	44.44%		
LGS	9	4	44.44%		
OLE	1	1	100.00%		
Not classified as epilepsy syndrome	31	19	61.29%		
**Drug use**					
Monotherapy	9	8	88.88%	3.112	0.078
Combination therapy	70	41	58.57%		

*BECT, Benign childhood epilepsy with centrotemporal spikes; ESES, Electrical status epilepticus during sleep; FLE, Frontal lobe epilepsy; CSWS, Cerebral Salt Wasting Syndrome; TLE, Temporal lobe epilepsy; LGS, Lennox-Gastaut Syndrome; OLE, Occipital lobe epilepsy*.

### Treatment response rates for different seizure types and epilepsy syndromes

Among the types of epilepsy, the effective rates of focal seizures and generalized seizures were 60.38 and 65.38%, respectively, and there was no statistical difference between the two groups (*P* > 0.05). Among the epilepsy syndromes, the categories with higher treatment efficacy were benign childhood epilepsy with centrotemporal spikes (BECT), BECT combined with electrical status epilepticus during sleep (ESES) and frontal lobe epilepsy (FLE), with treatment efficacy rates of 88.89, 72.73, and 66.67%, respectively (Table 2).

### Comparison of effective rates of monotherapy and combination therapy

Nine patients were treated with perampanel monotherapy, eight of them were effective, and the effective rate was 88.89%. Combined treatment of 70 cases, the number of effective cases was 41 cases, and the effective rate was 58.57%. There was no statistical difference in the effective rate between the two groups (*P* > 0.05; Table 2)

### Adverse reaction monitoring

**Figure 1:**
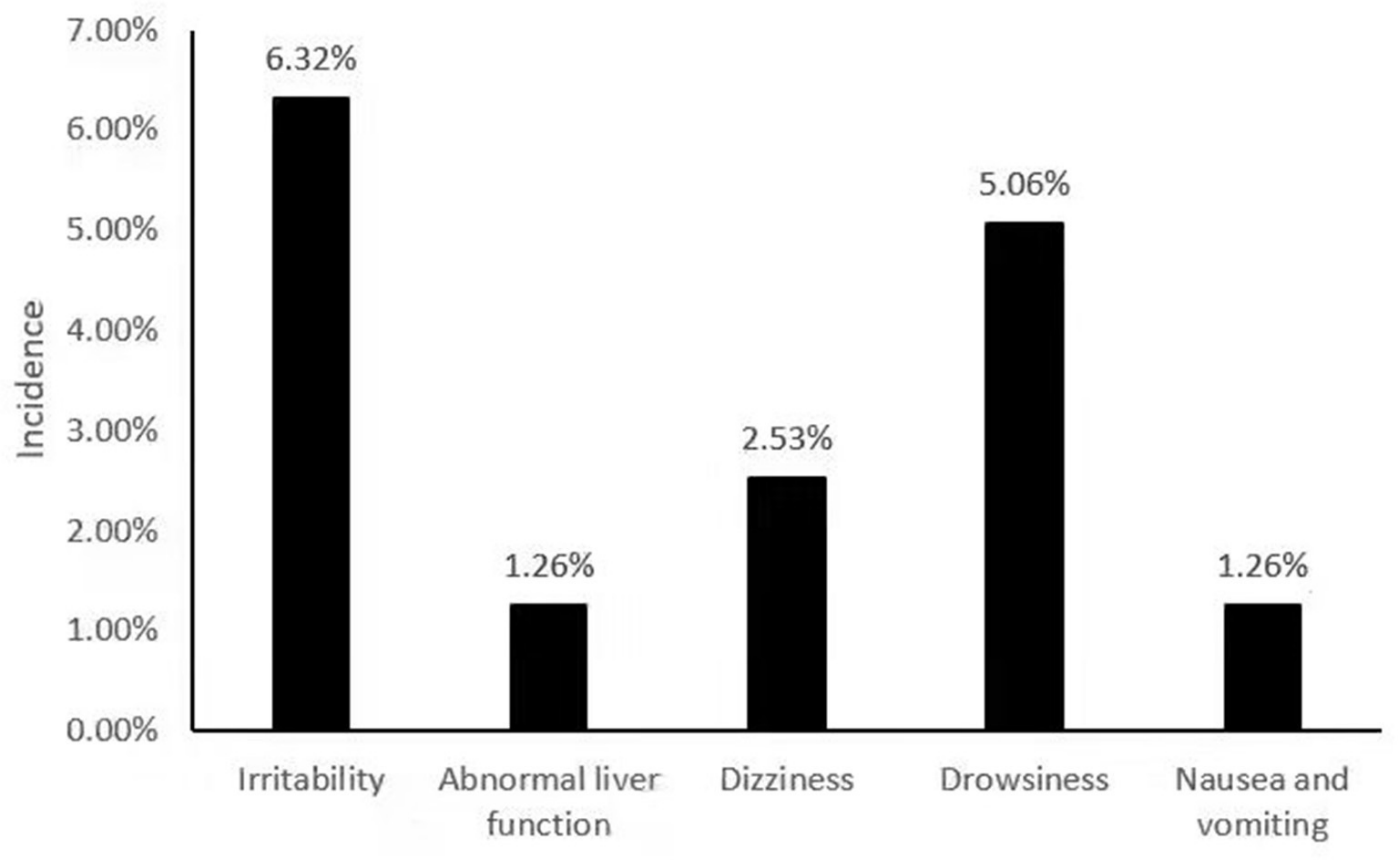
Perampanel adverse reaction monitoring.

Among 79 patients taking perampanel, adverse reactions were detected in 13 patients, and the incidence of adverse reactions was 16.45%. The main adverse reactions were irritability in five cases, the incidence rate was 6.32%; drowsiness in four cases, the incidence rate was 5.06%; dizziness in two cases, the incidence rate was 2.53%; abnormal liver function in one case, the incidence rate was 1.26%; Nausea and vomiting occurred in one case, with an incidence rate of 1.26% (Figure 1).

## Discussion

In the pathogenesis of epilepsy, glutamate, as the main excitatory neurotransmitter in the nervous system, binds to its ionotropic receptor [α-Amino-3-hydroxy-5-methyl-4-isoxazolepropionic acid (AMPA)] to cause Neurons depolarize, lowering the seizure threshold of epilepsy, thereby causing seizures (7). This mechanism has become a new target for novel antiepileptic drugs. Perampanel, a third-generation novel antiepileptic drug (AED), is the first highly selective, non-competitive AMPA receptor antagonist for the treatment of epilepsy. It inhibits excitatory neurotransmission by targeting postsynaptic glutamate activity, thereby exerting antiepileptic effects (7–10). Since its listing, it has been used in the treatment of childhood epilepsy in many countries. In some retrospective clinical studies, perampanel has been found to have a good therapeutic effect on various types of epilepsy, including focal seizures, focal secondary generalized seizures, primary Generalized tonic-spasmodic seizures and some special types of epilepsy syndromes, etc., and long-term adjuvant therapy has not caused new safety and tolerability problems (6, 11).

This study observed the treatment effect of perampanel in children with epilepsy and found that the overall effective rate of perampanel in the treatment of epilepsy was 62.03%, which was similar to the 59.6 and 67.9% effective rates of other studies (6). The patients were divided into groups according to age and divided into groups <4 years old, 4–12 years old and >12 years old. There was no difference in the effective rate of perampanel among the groups, indicating that perampanel has a good therapeutic effect in children of all ages. In a foreign study, it was confirmed that there was no difference in the effective rates of different types of monotherapy (initial monotherapy and replacement monotherapy) in patients with a younger age from 1 to 57 months (12). According to the analysis of seizure types, the effective rates of focal seizures and generalized seizures were both above 60%, which was similar to the findings of Villanueva and Swiderska et al. (12–14). According to the classification of epilepsy syndromes, perampanel had higher effective rates for BECT, BECT combined with ESES, FLE, temporal lobe epilepsy (TLE) and Cerebral Salt Wasting Syndrome (CSWS), all of which were more than 50%, and relatively low for Dravet and Lennox-Gastaut Syndrome (LGS). Occipital lobe epilepsy (OLE) was not discussed because of the small number of cases. In some small sample clinical studies and case reports involving children, perampanel may also be effective in treating pediatric Dravet syndrome, LGS and other refractory epilepsy (15). A retrospective study of epilepsy patients under 18 years of age included five patients with Dravet syndrome with a response rate of 80%, and a prospective study of children and adolescents included 13 patients with LGS with a response rate of 69.2%. These two studies have small sample sizes and need to be supported by more clinical results (16).

Our results showed that there was no significant difference in the effective rate between monotherapy and combination therapy. Unfortunately, there is a gap between the sample sizes of the two groups (9 and 70), which weakens the consistency of the results. In a phase III clinical study in Japan and South Korea, the effective rate of pirenpanet monotherapy was 63% (17). In a phase IV clinical trial in the United States, the effective rate of combined therapy was 76.5%, slightly higher than the average effective rate of 62.02% in this study (18). Recent literature suggested that whether in children or adult patients, non-high-dose P drugs combined with 1 or 2 ASMs achieved good effectiveness and has been further verified in the long-term 36–48 months of follow-up (19). Liver enzyme inhibitors, such as LEV and VPA, were not associated with changes in drug metabolic rate or blood concentration in patients treated with perampanel. Liver enzyme inducers, such as OXC, phenytoin sodium, CBZ, phenobarbital, etc., can accelerate the metabolism and reduce the plasma concentration of perampanel. This weakening can be addressed by speeding up administration or increasing the concentration of perampanel (20).

During the use of perampanel, we detected that the incidence of adverse reactions in patients was 16.45%. The main adverse reactions were: irritability, drowsiness, dizziness, nausea, vomiting and abnormal liver function. In a study of 493 epilepsy patients (including adolescents, adults, and the elderly), 28.2% of 243 patients who added perampanel had adverse reactions, mainly dizziness, irritability, balance disorders and weight gain (21). All adverse reactions were within the controllable range, and there was no situation that seriously affected the patient's vital signs or caused the recurrence and aggravation of epilepsy.

There are still some limitations in our study. First of all, our study is a retrospective study and did not include a control group, and the evidence level is low. In addition, the sample size is small, leading to a large gap in sample size between subgroups (such as different treatment methods), which weakens the consistency of conclusions. Large-scale prospective randomized controlled trial is still needed to verify this conclusion, which is also the focus of our next step.

## Conclusion

In conclusion, the new type of antiepileptic drug, perampanel has high efficacy, safety and tolerability during clinical use. But there will also be a certain degree of controllable adverse reactions. The drug remains a reliable option for long-term use in the treatment of epilepsy.

## Data availability statement

The original contributions presented in the study are included in the article/supplementary material, further inquiries can be directed to the corresponding author.

## Ethics statement

The studies involving human participants were reviewed and approved by the Second Affiliated Hospital of Xi'an Jiaotong University. Written informed consent to participate in this study was provided by the participants' legal guardian/next of kin.

## Author contributions

DL and TS contributed to the conception and design of the study. SH and XW contributed to the acquisition of data. SH and LY contributed to the analysis of data. DL wrote the manuscript. TS revised the manuscript. All authors participated in the clinical practice, including diagnosis, treatment, consultation and follow up of patients, and approved the final version of the manuscript.

## Funding

This study was supported by Key R&D program of Shaanxi Province (No. 2021SF-087).

## Conflict of interest

The authors declare that the research was conducted in the absence of any commercial or financial relationships that could be construed as a potential conflict of interest.

## Publisher's note

All claims expressed in this article are solely those of the authors and do not necessarily represent those of their affiliated organizations, or those of the publisher, the editors and the reviewers. Any product that may be evaluated in this article, or claim that may be made by its manufacturer, is not guaranteed or endorsed by the publisher.
